# Identification of photosynthetic parameters for superior yield of two super hybrid rice varieties: A cross-scale study from leaf to canopy

**DOI:** 10.3389/fpls.2023.1110257

**Published:** 2023-02-14

**Authors:** Yonghui Pan, Yiwen Cao, Yixiao Chai, Xusheng Meng, Min Wang, Guanjun Wang, Shiwei Guo

**Affiliations:** ^1^ Jiangsu Provincial Key Lab for Organic Solid Waste Utilization, National Engineering Research Center for Organic-based Fertilizers, Jiangsu Collaborative Innovation Center for Solid Organic Waste Resource Utilization, Nanjing Agricultural University, Nanjing, Jiangsu, China; ^2^ Center of Agricultural Green Development Promotion, Fuyang, Anhui, China

**Keywords:** yield, photosynthetic capacity, super rice, canopy photosynthesis, leaf nitrogen concentration

## Abstract

Enhancing photosynthetic capacity is widely accepted as critical to advancing crop yield. Therefore, identifying photosynthetic parameters positively related to biomass accumulation in elite cultivars is the major focus of current rice research. In this work, we assessed leaf photosynthetic performance, canopy photosynthesis, and yield attributes of super hybrid rice cultivars Y-liangyou 3218 (YLY3218) and Y-liangyou 5867 (YLY5867) at tillering stage and flowering stage, using inbred super rice cultivars Zhendao11(ZD11) and Nanjing 9108 (NJ9108) as control. A diurnal canopy photosynthesis model was applied to estimate the influence of key environmental factors, canopy attributes, and canopy nitrogen status on daily aboveground biomass increment (AM_DAY_). Results showed that primarily the light-saturated photosynthetic rate at tillering stage contributed to the advancing yield and biomass of super hybrid rice in comparison to inbred super rice, and the light-saturated photosynthetic rate between them was similar at flowering stage. At tillering stage, the higher CO_2_ diffusion capacity, together with higher biochemical capacity (i.e., maximum carboxylation rate of Rubisco, maximum electron transport rate (*J*_max_), and triose phosphate utilization rate) favored leaf photosynthesis of super hybrid rice. Similarly, AM_DAY_ in super hybrid rice was higher than inbred super rice at tillering stage, and comparable at flowering stage partially due to increased canopy nitrogen concentration (SLN_ave_) of inbred super rice. At tillering stage, model simulation revealed that replacement of *J*_max_ and *g*_m_ in inbred super rice by super hybrid rice always had a positive effect on AM_DAY_, and the averaged AM_DAY_ increment was 5.7% and 3.4%, respectively. Simultaneously, the 20% enhancement of total canopy nitrogen concentration through the improvement of SLN_ave_ (TNC-SLN_ave_) resulted in the highest AM_DAY_ across cultivars, with an average increase of 11.2%. In conclusion, the advancing yield performance of YLY3218 and YLY5867 was due to the higher *J*_max_ and *g*_m_ at tillering stage, and TCN-SLN_ave_ is a promising target for future super rice breeding programs.

## 1 Introduction

Rice (*Oryza sativa* L.), one of the most important food crops in the world, provides 35–60% of calories for about 3 billion people. It has been estimated that the global population will increase to nearly 10 billion by 2050, requiring a 70% -100% increase in the yield of major food crops (Tilman et al., 2011; Tilman and Clark, 2015). However, the year-on-year increase in the yield of rice in many parts of the world has plateaued, while the potential for expanding arable land is limited (Simkin et al., 2019). Clearly, it becomes an urgent task to improve rice yield potential per harvested area, while improving leaf photosynthetic efficiency is regarded as a vital approach (Zhu et al., 2010; Faralli and Lawson, 2020). Some strategies have been proposed for improving leaf photosynthesis, such as engineering more efficient ribulose-1,5-bisphosphate carboxylase/oxygenase (Rubisco) (Whitney and Sharwood, 2008), increasing the recovery rate of photosystem II from the photo-protected state (Zhu et al., 2004), introducing the CO_2_ concentration mechanism into crops (Price et al., 2008), facilitating synchronous stomatal conductance response with mesophyll demands for CO_2_ (Mcausland et al., 2016). However, these manipulations would require research efforts of decades as stated by Long et al. (2016). Recently, Yoon et al. (2020) provided evidence that an engineered increase in Rubisco production increased rice yield and nitrogen use efficiency for biomass production under field experiments, which is a promising start and could translate into other genotypes and environments in the future.

Considering complicated constraints of engineering, natural variation in the photosynthetic capacity of rice cultivars can be explored by identifying superior cultivars and traits and integrating them into breeding projects for enhancement of rice yield potential, which is the most efficient approach in short term (< 5 years) as indicated by Parry et al. (2011). However, improvement in leaf-level photosynthesis did not necessarily increase plant biomass or crop yield. Driever et al. (2014) found natural variations in photosynthetic capacity by testing 64 wheat cultivars, while no significant relationship between photosynthetic capacity at the leaf level and yield was found. As pointed out by Yin and Struik (2015), these analyses focused primarily on potential maximum conversion efficiency, with little quantitative allowance for the case of light saturation of photosynthesis (*A*_sat_). More importantly, they paid little attention to the scaling up of the evaluation at the leaf level into quantitative information at the canopy level in space. Leaf photosynthesis is determined by CO_2_ diffusion capacity including stomatal conductance (*g*_s_) and mesophyll conductance (*g*_m_) and biochemical factors (maximum Rubisco carboxylation rate, *V_cmax_*; maximum electron transport rate, *J*_max_) (Flexas et al., 2008; Flexas et al., 2012). While canopy photosynthesis is more related to crop yield, which is determined by canopy structure, canopy nitrogen distribution, leaf-level photosynthesis, and its interaction with environment parameters (solar radiation, air temperature, vapour pressure deficit, and atmospheric CO_2_ concentration) (Yin and Struik, 2015; Peng, 2000). A concise procedure for scaling up from instantaneous leaf assimilation to daily canopy photosynthesis (*A*_can,day_) and then subsequently to total biomass production over a crop cycle is critical for selecting crop varieties with higher canopy photosynthesis and thereby the substantial crop yield. While the measurement of canopy photosynthesis directly is difficult, this can be addressed by running numerical simulations using canopy photosynthesis models instead.

So far, many canopy photosynthesis models have been developed. The big leaf model (Running and Coughlan, 1988; Sellers et al., 1992), sunlit-shaded model (Gu et al., 2014; Wu et al., 2018), multi-layer model (Amthor, 1994; De Pury and Farquhar, 1997), 3D canopy architecture model (Song et al., 2013; Shi et al., 2019) were used to simulate canopy light distribution with different levels of complexity and accuracy. For rice, a 3D canopy architecture model (Song et al., 2013; Shi et al., 2019) was used to estimate canopy photosynthesis, which focused on the impacts of manipulation of canopy structure (stem height, leaf width, leaf angle) on canopy CO_2_ assimilation. However, leaf photosynthesis was simulated *via* light response curves rather than photosynthetic mechanistic models and they ignored environment fluctuations (atmosphere CO_2_ concentration, vapour pressure deficit, air temperature). Gu et al. (2014) used the mechanistic model connected with GECROS to analyze photosynthetic manipulations on rice biomass production based on quantitative trait loci related to different photosynthetic parameters. This was inspiring because with the rapid advances of plant genomics research, the modeling work could potentially help guide progress in photosynthetic enhancement for field crop improvement through crop breeding, engineering, and also precision agriculture. The daily canopy photosynthesis-stomatal conductance model (DCaPS) developed by Wu et al. (2018) was a standalone diurnal canopy photosynthesis simulator, which upscaled the biochemical models of photosynthesis to a canopy level for simulating canopy CO_2_ assimilation of crop over a day. It incorporated the influences of total canopy nitrogen concentration (TCN), canopy leaf area index (LAI_can_), average canopy nitrogen concentration per unit area (SLN_ave_), canopy photosynthetic nitrogen extinction coefficient (*K*_N_), *V_cmax_*, *J*_max,_ and *g*_m_ on canopy photosynthesis. In this sense, this model adopted in rice research will help identification of efficient approaches to improve rice canopy photosynthesis and grain yield.

Since 1996, the establishment of the “super rice” mega-project had constantly achieved leaps in the yield potential of rice in China. Up to 2021, 135 “super” rice varieties have been identified by the Ministry of Agriculture (http://www.ricedata.cn/), and some cultivars achieved grain yields of more than 12 t ha^-1^ (Peng et al., 2008). To date, the comparison in the grain yield was primarily conducted among the super hybrid, inbred, and ordinary hybrid rice to clarify the yield advantage of “super” rice (Zhang et al., 2015; Liu et al., 2018; Li et al., 2019). However, some studies show that there are substantial variations in grain yield (Chen et al., 2014), rice architectures (Zhang et al., 2014), leaf photosynthetic properties (Wei et al., 2018), and nutrient utilization (Ahmed Jewel et al., 2019) among the super high yielding varieties. In this context, to break the yield ceiling of rice production, it was essential to establish the mechanism underlying the yield advantage and photosynthetic capacity in super hybrid rice and inbred super rice. Our previous study found that Yliangyou 3218 (YLY 3218) achieved a high yield due to its improved radiation use efficiency (Pan et al., 2020). Therefore, a comparison in the yield performance and leaf-level photosynthesis was performed between two super hybrid rice Y-liangyou 3218 (YLY3218) and Y-liangyou 5867 (YLY5867), and two inbred super rice Zhendao11 (ZD11) and Nanjing9108 (NJ9108) at two growth stages (tiller stage and flowering stage) in the present study. Then simulated daily aboveground biomass increment (AM_DAY_) *via* DCaPS. Furthermore, based on the canopy photosynthetic model, we adopted single factor enhancement or substitution analysis method. These systematic analyses would enable us to identify differences in crop biomass production patterns and leaf photosynthesis, and the key factors affecting canopy photosynthetic rate at different growth stages.

## 2 Materials and methods

### 2.1 Plant material and growth conditions

The field experiment was conducted in Rugao County, Jiangsu Province, China (32°26′24″N, 120° 29′24″E). The upper soil (0-20 cm) chemical properties were as follows: pH 7.54, organic matter 20.2 g kg^-1^, total nitrogen 1.97 g kg^-1^, Olsen-P 10.8 mg kg^-1^, NH_4_OAc-K 92.0 mg kg^-1^. Two different types of super rice cultivars, namely YLY3218 and YLY5867 as super hybrid rice cultivars and ZD11 and NJ9108 as two inbred super rice cultivars were used in the study. Seeds for the cultivars were sown on seedbeds after germination on May 20^th^ of 2020; uniform seedlings for each cultivar were transplanted on June 20^th^ of 2020 with a hill spacing of 25×13 cm (two plants per hill). The experiment was performed in a randomized block design consisting of three replicates, and the plot area of each treatment was 25 m^2^. Nitrogen fertilizer (urea) was set as 90 kg nitrogen ha^-1^ and applied on four dates: 40% as basal fertilizer, 20% at the mid-tillering stage, 20% at the panicle initiation stage, and 20% at the spikelet differentiation stage. Phosphorus (calcium superphosphate, 75 kg P_2_O_5_ ha^-1^) and potassium (potassium chloride, 90 kg K_2_O ha^-1^) were supplied with equal amounts for each treatment at the basal and panicle initiation stage, and the proportion of phosphorus or potassium was 50% and 50% at these two stages. Weeds, pests, and diseases were controlled periodically with herbicides, insecticides, and fungicides. The weather data during rice growth period, including the air temperature, precipitation, and photosynthetically active radiation, was recorded by a meteorological station located next to the field (**Figure S1**).

### 2.2 Gas exchange and fluorescence measurements

Leaf photosynthetic gas exchange parameters were measured using a portable, open-circuit, infrared gas analysis system (LI-6400XT, LI-COR Inc., Lincoln, NE, USA) equipped with an integrated fluorescence leaf chamber (LI-6400-40) at tillering stage (July 25^th^, 2020) and flowering stage (September 1^st^, 2020). At least six newly expanded leaves for each cultivar were selected for simultaneous measurement of gas exchange and chlorophyll fluorescence from 9:00 to 15:00. Steady-state photosynthesis under light-saturating conditions was reached by using a photosynthetic photon flux density of 1500 μmol m^-2^ s^-1^. Besides, we set leaf temperature as 35 ± 0.1 °C, reference CO_2_ concentration as 400 μmol mol^−1^, air flow rate as 500 μmol s^-1^, and relative humidity as 55-60%; leaves were acclimated in the leaf chamber for 15-20 min to a steady-state; then the gas exchange parameters such as *A*_sat_, g_s_, and intercellular CO_2_ concentration (*C*_i_) and steady-state fluorescence yield (*F*_s_) and maximum fluorescence (*F*_m_ *′*) with a light saturating pulse (0.8 s) of approximately 8000 μmol photons m^-2^ s^-1^ were recorded.

The effective quantum efficiency of photosystem II (Φ_PSII_) was quantified as:


(1) $$\Phi_{PSII}=\left(F_{m}{\;}^{\prime }-F_{s}\right)/F_{m}{\;}^{\prime }$$

The potential electron transport rate (*J*) was calculated as:


(2) $$J=\Phi_{PSII}\times PPFD\times \alpha \times \beta$$

where the PPFD is the photosynthetic photon flux density; α is the leaf absorption and β is the proportion of quanta absorbed by PSII. Values of α and β were determined following the method presented by Yin et al. (2009). Briefly, the leaf photosynthetic light response curve was measured under the condition of 2% oxygen concentration; the light levels were changed to 200, 150, 75, 50, and 30 μmol m^-2^ s^-1^. The parameter (Φ_PSII_ × PPFD/4) obtained in curve is linear related to the net photosynthetic rate, whose slope is the product of α and β.

### 2.3 CO_2_ response curves

CO_2_ response curves (*A*/*C*_i_ curves) were measured on leaves which were used for measurements of gas exchange and chlorophyll fluorescence (at least four leaves per cultivar). For each *A*/*C*_i_ curve, leaves were acclimated in the leaf chamber for at least 30 min; then the CO_2_ concentration decreased stepwise from 400 to 300, 250, 200, 100 and increased from 100 to 400, 450, 500, 550, 650, 800 and 1000 μmol mol^-1^. Data were recorded when the gas exchange parameters were stabilized at a given CO_2_ concentration. Chloroplast CO_2_ concentration (*C*_c_) and *g*_m_ were calculated as described by Sharkey et al. (2007).


(3) $${\text{g}}_{\text{m}}=\frac{\text{A}}{{\text{C}}_{\text{i}}-\frac{{\text{Γ}}^{*}{\text{(J+8(A+R}}_{\text{d}}\text{))}}{\text{J}-{\text{4(A+R}}_{\text{d}})}}\;\;\;\;\;\;\;\;\;\;\;\;$$

(4) $$\;\;\;\;\;{\text{C}}_{\text{c}}{\text{=C}}_{\text{i}}-\frac{\text{A}}{{\text{g}}_{\text{m}}}\;\;\;\;\;\;\;\;\;$$

Γ^*^ is the CO_2_ compensation point in the absence of mitochondrial respiration, which was set as 40.0 μmol m^-2^ s^-1^. *R*_d_ is the mitochondrial respiration rate in the light. In this study, *R*_d_ was determined as the vertical intercept of the fitting line between Φ_PSII_× PPFD/4 and net photosynthetic rate as mentioned above.

Accordingly, *A*/*C*_i_ curves were transferred to *A*/*C*_c_ curves, which were used for the estimation of *V_cmax_*, *J_max_*, and triose phosphate utilization rate (TPU).

### 2.4 Biomass, leaf area index, leaf nitrogen concentration, grain yield, and leaf inclination angle (β)

After measuring the photosynthetic parameters, five plants for each cultivar were randomly sampled. Firstly, plants were separated into the latest fully expanded leaf, other leaves, stem, and root. The newly expanded leaves were digitally scanned (ES-1200C scanner; Epson, Long Beach, CA, USA), and the area was computed by Image J software (National Institutes of Health, Bethesda, MD, USA). LAI was the product of the total leaf area per plot of plant and transplanting density. The samples were then desiccated at 105°C for 30 min and dried at 70°C to constant weight, followed by weighing and milling. For leaf nitrogen content, the milled latest fully expanded leaf samples were digested with H_2_SO_4_-H_2_O_2_ at 280°C and the nitrogen concentration of each sample was determined with a continuous flow analyzer (AA3; Seal Analytical, Inc., Southampton, UK). The leaf area, biomass, and nitrogen concentration of the top leaves and the other leaves were measured following the method presented above. The SLN_ave_ (g m^-2^) was calculated as:


$${\text{SLN}}_{\text{ave}}=\frac{{\text{N}}_{\text{o}}{*\text{DM}}_{\text{o}}{+\text{N}}_{\text{other}}{*\text{DM}}_{\text{other}}}{{\text{LA}}_{\text{o}}{*\text{LA}}_{\text{other}}}{*(\text{DM}}_{\text{o}}{+\text{DM}}_{\text{other}})$$

where the LA_o_ is the area of the top leaves (m^2^), the LA_other_ is the area of the other leaves (m^2^), the DM_o_ is the biomass of the top leaves (g), the DM_other_ is the biomass of the other leaves (g), N_o_ is the nitrogen content of the top leaves (%) and N_other_ is the nitrogen content of the other leaves (%).

For both growth stages, the plant canopy analyzer (LAI-2200C, LI-COR Inc., Lincoln, NE, USA) was used to determine the β for each cultivar. Briefly, in each plot, the analyzer was positioned at the top of the canopy to record the incident solar radiation 5 times and then 20-30 times at the bottom of the canopy. The instrument will automatically obtain the β of the canopy.

At the harvest stage, brown grain yield was estimated by a 6 m^2^ area plants for each plot (super hybrid rice, 16 October; inbred super rice, 28 October) and adjusted to a 14% water content.

### 2.5 Description of DCaPS

DCaPS developed by Wu et al. (2018) was used for simulating daily canopy CO_2_ assimilation (from sunrise to sunset) of rice. A schematic diagram of this model is shown in **Figure S2**, and detailed information about model parameters and equations is provided in **Tables S1–S3**. Model inputs are composed of environment, canopy architecture, canopy nitrogen status, CO_2_ diffusion, photosynthetic and temperature response parameters, which is listed in **Table S3**. Model outputs are diurnal environment variables, diurnal canopy photosynthesis. Environment parameters in the form of hourly values of incident solar radiation, air temperature (T_a_, an approximate value for leaf temperature), and air vapour pressure deficit for one day were derived from daily values. The LAI_can_ was split into sunlit and shade fractions by a single-layer sunlit-shade leaves modeling approach as described by De Pury and Farquhar (1997), and then the amount of photosynthetically active radiation including direct and diffuse solar radiation intercepted by each fraction was determined. Canopy nitrogen distribution (SLN_ave_; nitrogen concentration per unit leaf area at the top of the canopy, SLN_top_) was used to estimate daily nitrogen status for sunlit and shaded leaves followed by previous crop model (Hammer et al., 2010). The key photosynthetic parameters (the maximum carboxylation rate of Rubisco at 25°C, *V_cmax25_*; maximum electron transport rate at 25°C, *J_max25_*; mesophyll conductance at 25°C, *g*_m25_) were used to derive the slope of linear relationship between *V_cmax_* per leaf area at 25°C and nitrogen (χ_v_), and the slope of linear relationship between *J_max_* per leaf area at 25°C and nitrogen (χ*_J_*). Alternatively, hourly values of CO_2_ assimilate rate (minimum value of *A_c_* and *A_j_*, **Figure S1**) of sunlit and shaded leaves were determined after combining with nitrogen status, CO_2_ diffusion models and temperature adjustment based on photosynthetic parameters following Wu et al. (2018). Finally, *A*_can,day_ was determined from integration of CO_2_ assimilation rate across leaf fractions and time. The AM_DAY_ was calculated as:


$${\text{AM}}_{\text{DAY}}{\text{=A}}_{\text{can,day}}*44*0.41*0.85$$

where 44 is the molecular weight of CO_2_, 0.85 represents the dry matter distribution coefficient of the aboveground at tillering stage and flowering stage (dimensionless), and 0.41 was introduced as a conversion factor that accounts for the loss of CO_2_ assimilation (g biomass g^-1^ CO_2_), as described by Sinclair and Horie (1989).

### 2.6 Simulated scenarios, input variables, and validation simulations

Simulation of daily canopy photosynthesis was conducted for each treatment with its specific input parameters as listed in **Table S3**. To explore the most efficient target to improve daily canopy photosynthesis, canopy architecture parameters (LAI_can_, β), canopy nitrogen status parameters (TNC; SLN_ave_, *K*_N_) and photosynthetic parameters (*V_cmax_*, *J_max,_* and *g*_m_) were enhanced by 20% increment of associated model parameters. And the simulation results with adjusted parameters were compared with those of original simulations. The increment of LAI_can_ or SLN_ave_ resulted in TNC. Therefore, in the current simulation, TNC increment was through an improvement in one of these two factors and the other one keeps unchanged. When increasing LAI_can_ (SLN_ave_) by 20%, alternatively, SLN_ave_ (LAI_can_) should decrease by 20%, and the ratio of SLN_ave_ to SLN_top_ (SLN_ratio_top_) was also recalculated to ensure TNC and *K*_N_ were kept constant. For the improvement of *K*_N_, *V_camx_*, *J_max,_* and *g*_m_, *K*_N_, slope of linear relationship between *V_cmax_* per leaf area at 25°C and nitrogen (χ_v_), slope of linear relationship between *J_max_* per leaf area at 25°C and nitrogen (χ*_J_*) and g_m_ were directly increased by 20% without any other changes.

To verify the factors determining the difference in *A*_can,DAY_ between super hybrid rice and inbred super rice, every single parameter of inbred super rice was replaced by that of super hybrid rice, and the rules of parameter adjustment are the same as mentioned above.

### 2.7 Statistical analysis

Analysis of variance (*ANOVA*) was used to analyze the data, and the significant difference between cultivars or nitrogen treatments within a cultivar was accessed by the least significant difference (LSD) test (*P<* 0.05). Simulation of daily canopy photosynthesis was conducted by Visual Studio 2019 (Microsoft Corporation, Redmond, WA, USA) with application of the source code developed by Wu et al. (2018) which is available at https://github.com/QAAFI/DCaPS.git.

## 3 Results

### 3.1 Grain yield and biomass

**Figure 1:**
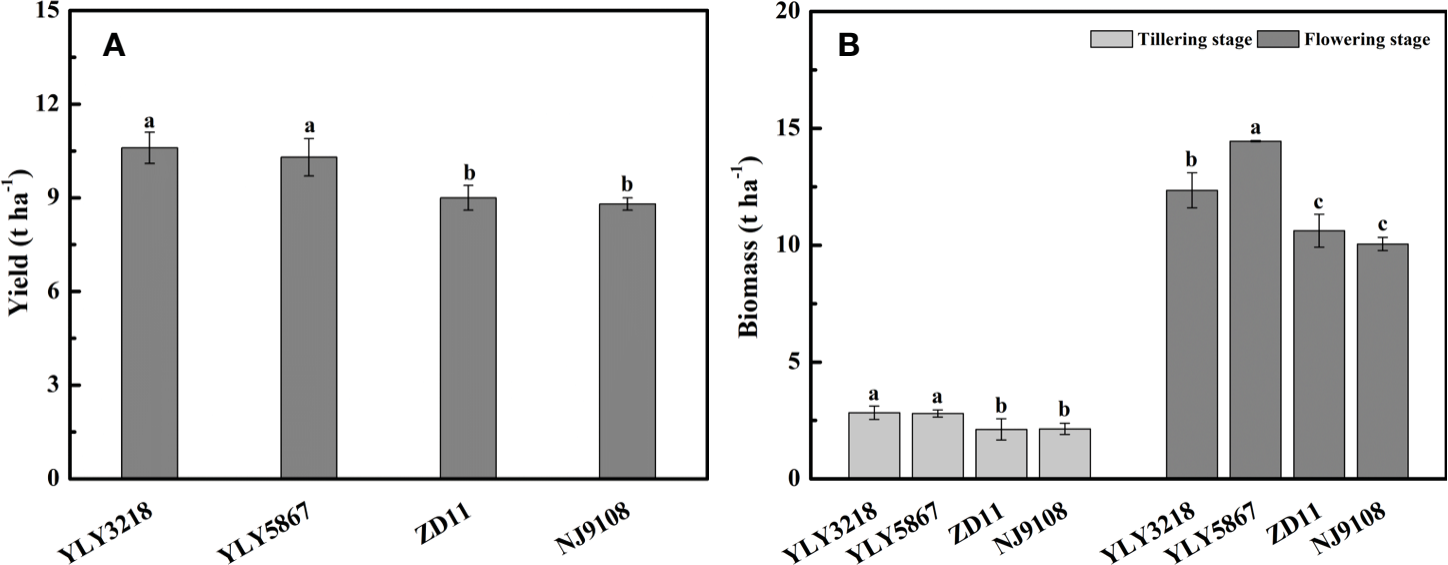
Yield **(A)** and total biomass **(B)** of different rice cultivars during tillering and flowering stage. Different letters mean significant differences (*P*< 0.05) among rice cultivars during the same growth stage.

Averaged across the cultivars, grain yield of super hybrid rice was significantly higher than that of the inbred super rice (**Figure 1A**). The grain yield of super hybrid rice was 10.6 t hm^-2^ (YLY3218) and 10.3 t hm^-2^ (YLY5867), respectively, while the inbred super rice had a lower grain yield than YLY3218 by 15.1% (ZD11) and 17.0% (NJ9108). In accordance with the grain yield, the super hybrid rice had a consistently higher amount of biomass than the inbred super rice throughout the tillering stage and flowering stage (**Figure 1B**).

### 3.2 A_sat_, leaf nitrogen content per area, and leaf CO_2_ diffusion parameters

**Figure 2:**
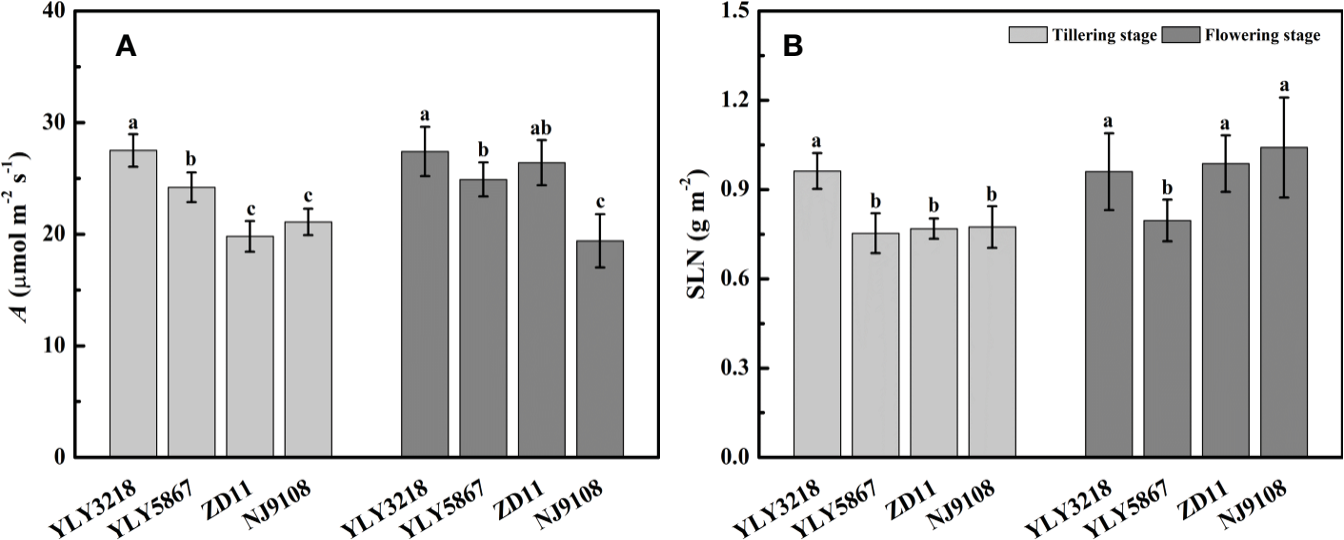
Net photosynthesis rate under saturated irradiance (*A*_sat_) **(A)** and nitrogen concentration per leaf area (SLN) **(B)** of the latest fully expanded leaves for different rice cultivars during tillering and flowering stage. Different letters mean significant differences (*P*< 0.05) among rice cultivars during the same growth stage.

*A*_sat_ of YLY3218 was significantly higher than that of inbred super rice (ZD11 and NJ9108) at tillering stage (**Figure 2A**); whereas it was comparable to ZD11 at flowering stage. Interestingly, there was no consistent difference in SLN at both stages between the super hybrid rice and the inbred super rice (**Figure 2B**). We further studied the photosynthetic parameters of each cultivar at both stages. Coincidence with the *A*_sat_, *g*_s_ and *g*_m_ of super hybrid rice were significantly higher than those of inbred super rice at tillering stage; however, those variations associated with cultivars disappeared during flowering stage (**Table 1**).

**Table 1:** CO_2_ diffusion parameters pf the latest fully expanded leaves for different rice cultivars during tillering stage and flowering stage.

Stage	Variety	*g*_s_ (mol m^-2^ s^-1^)	*C*_i_ (μmol mol^-1^)	*g*_m_ (mol m^-2^ s^-1^)	*C*_c_ (μmol mol^-1^)
Tillering	YLY3218	0.499 ± 0.037a	280 ± 5b	0.250 ± 0.024a	170 ± 7a
	YLY5867	0.485 ± 0.036a	293 ± 5a	0.180 ± 0.019b	153 ± 10b
	ZD11	0.344 ± 0.050b	284 ± 11ab	0.136 ± 0.022c	137 ± 11c
	NJ9108	0.364 ± 0.033b	284 ± 11ab	0.154 ± 0.018bc	149 ± 13bc
Flowering	YLY3218	0.450 ± 0.064a	272 ± 8b	0.287 ± 0.098ab	169 ± 20 b
	YLY5867	0.457 ± 0.031a	284 ± 6a	0.318 ± 0.071a	198 ± 25 a
	ZD11	0.462 ± 0.095a	278 ± 12ab	0.231 ± 0.024b	160 ± 14 bc
	NJ9108	0.316 ± 0.059b	277 ± 9ab	0.145 ± 0.038c	140 ± 27 c

g_s_: stomatal conductance; C_i_: intercellular CO_2_ concentration; g_m_: mesophyll conductance; C_c_: chloroplast CO_2_ concentration. Data are mean ± SD. Different letters mean significant differences (P< 0.05) among rice cultivars during the same growth stage.

At the tillering stage, *V*_cmax_, *J*_max_, and TPU were greater in super hybrid rice than in inbred super rice (**Table 2**). At flowering stage, *V*_cmax_ was significantly higher in YLY3218 (141.1 μmol m^-2^ s^-1^) than in the inbred super rice; however, there was no difference in the *V*_cmax_ between the cultivars. The *J*_max_ (221.7 μmol m^-2^ s^-1^) and TPU (13.3 μmol m^-2^ s^-1^) observed in YLY3218 were similar to ZD11, both of which were significantly higher than that in YLY5867 and NJ9108.

**Table 2:** Maximum carboxylation rate, maximum electron transfer rate and triose phosphate utilization rate of the latest fully expanded leaves for different rice cultivars during tillering and flowering stage.

Stage	Variety	*V_cmax_* (μmol m^-2^ s^-1^)	*J_max_* (μmol m^-2^ s^-1^)	TPU (μmol m^-2^ s^-1^)
Tillering	YLY3218	140.4 ± 15.8a	214.7 ± 11.3a	12.3 ± 0.62a
	YLY5867	152.3 ± 24.8a	185.2 ± 11.3b	11.4 ± 0.90a
	ZD11	90.0 ± 10.3b	156.8 ± 14.8c	9.08 ± 0.96b
	NJ9108	91.9 ± 8.7b	155.0 ± 9.8c	9.41 ± 0.96b
Flowering	YLY3218	141.1 ± 23.7a	221.7 ± 13.6 a	13.3 ± 0.64a
	YLY5867	122.3 ± 16.3ab	192.1 ± 14.6 b	11.8 ± 0.72b
	ZD11	113.2 ± 6.1bc	216.4 ± 7.7 a	12.8 ± 0.55a
	NJ9108	95.2 ± 5.9c	174.3 ± 13.9 b	11.4 ± 0.64b

V_cmax_, maximum carboxylation rate; J_max_, maximum electron transfer rate; TPU, triose phosphate utilization rate. Data are mean ± SD. Different letters mean significant differences (P< 0.05) among rice cultivars during the same growth stage.

### 3.3 LAI, β, and average canopy nitrogen concentration

**Figure 3:**
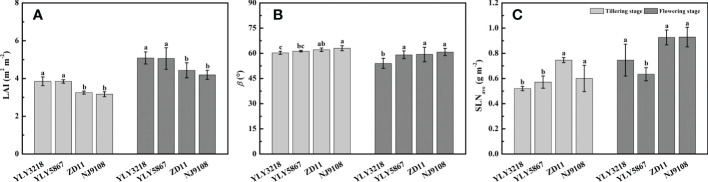
Leaf area index (LAI) **(A)**, leaf inclination angle (β) **(B)**, and average canopy nitrogen concentration per unit leaf area (SLN_ave_) **(C)** for different rice cultivars during tillering stage and flowering stage. Different letters mean significant differences (*P*< 0.05) among rice cultivars during the same growth stage.

Paralleled with the reductions in grain yield and biomass, a significant reduction in LAI of inbred super rice was found of 15.1%-17.7% at tillering stage and 14.2%-17.7% at flowering stage (**Figure 3A**). Generally, β of flowering stage was lower than that of tillering stage (**Figure 3B**). We noted that the β of super hybrid rice was not higher than that of inbred super rice. At both tillering stage and flowering stage, the inbred super rice had consistently the highest β value, comparable to YLY5867 but statically higher than YLY3218. There was no difference in the SLN_ave_ at tillering stage between the super hybrid rice and the inbred super rice (**Figure 3C**). While the SLN_ave_ value of inbred super rice was significantly higher than super hybrid rice at flowering stage, the maximal SLN_ave_ was reached in NJ9108 (**Figure 3C**).

### 3.4 AM_DAY_ simulated by improvement in targeted parameters

**Figure 4:**
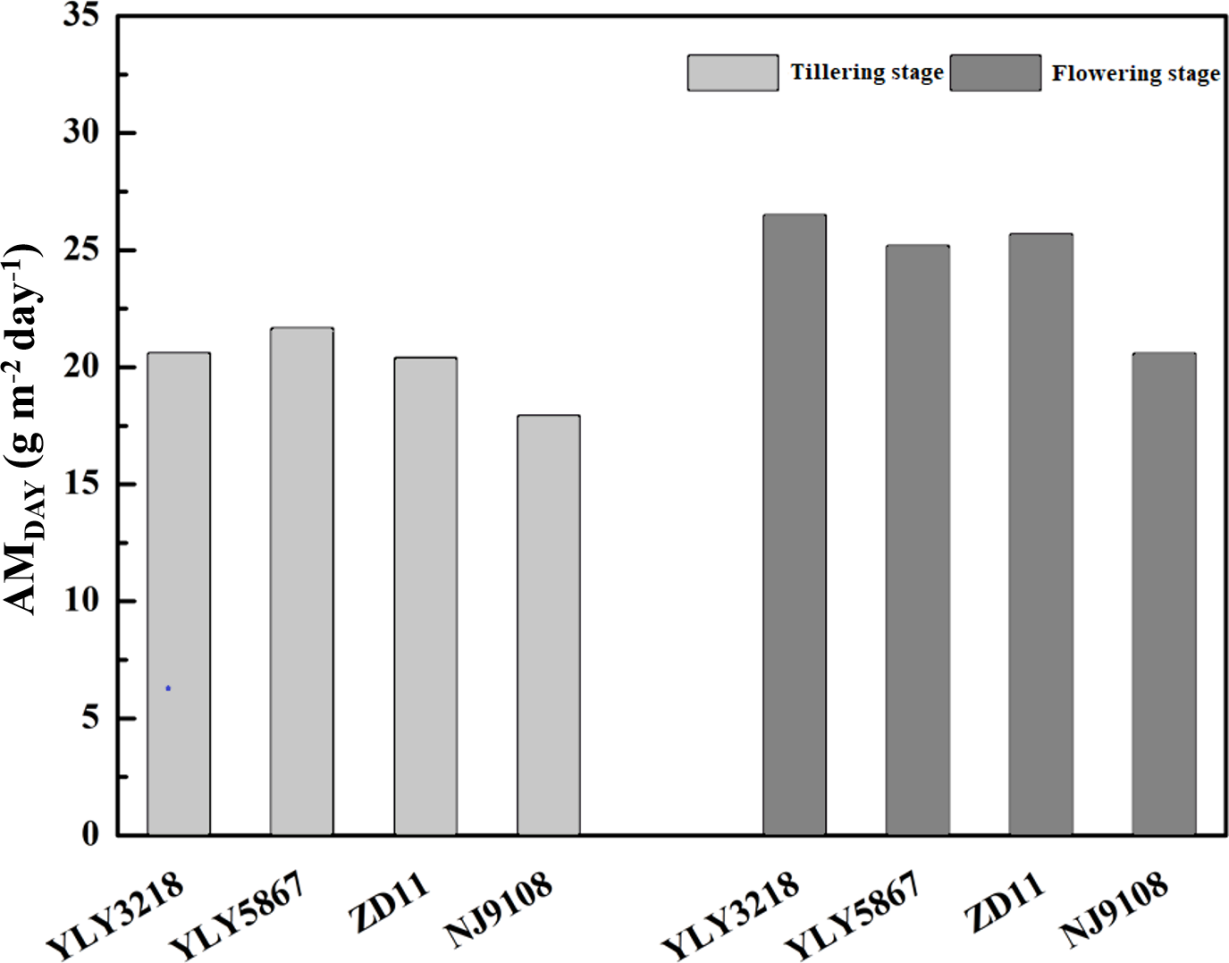
Daily aboveground biomass increment (AM_DAY_) simulated by observed parameters for different rice cultivars during tillering and flowering stage. Different letters mean significant differences (*P*< 0.05) among rice cultivars during the same growth stage.

**Figure 5:**
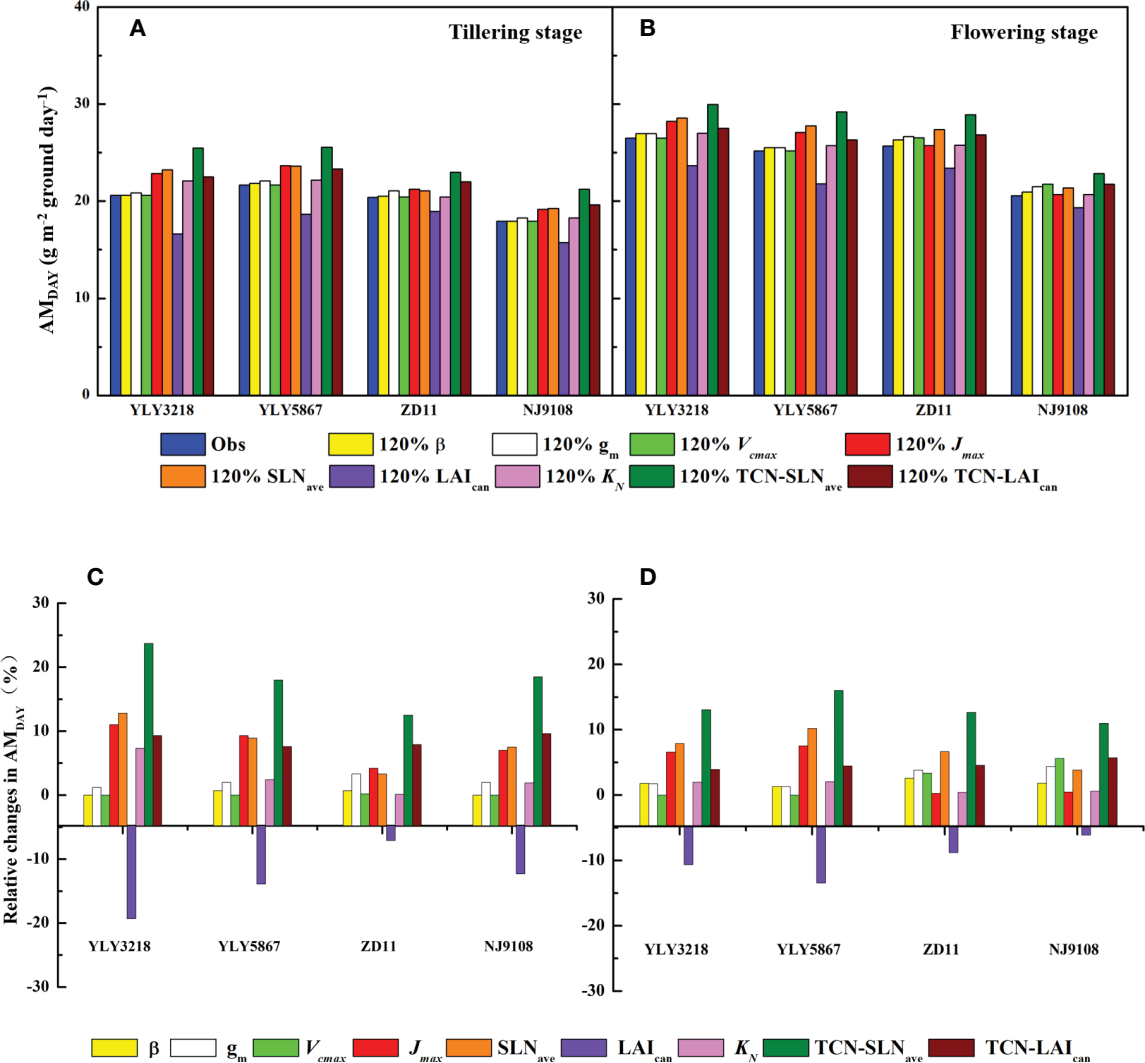
AM_DAY_ simulated by 20% enhancement in targeted parameters **(A, B)** and the corresponding percentage change **(C, D)** for different rice cultivars during tillering **(A, C)** and flowering stage **(B, D)**. Targeted parameters include canopy average leaf inclination relative to the horizontal (β), mesophyll conductance (*g*_m_), maximum carboxylation rate (*V*_cmax_), maximum electron transfer rate (*J*_max_), average canopy nitrogen concentration per unit leaf area (SLN_ave_), canopy leaf area index (LAI_can_), canopy photosynthetic nitrogen extinction coefficient (*K*_N_), total canopy nitrogen concentration through improvement in SLN_ave_ (TNC-SLN_ave_), total canopy nitrogen concentration through improvement in LAI_can_ (TNC-LAI_can_).

According to Wu et al. (2018), simulation of daily canopy photosynthesis for the hybrid super rice and the inbred super rice based on specific input parameters in the present study was done using DCaPS. At tillering stage, as shown in **Figure 4**, the values of AM_DAY_ based on the observed parameters ranged from 17.92 g m^-2^ to 21.66 g m^-2^. The super hybrid rice achieved a high compared to the inbred super rice. To find out the most promising target for enhancing AM_DAY_, 20% improvement of AMDAY value of different targets were used to complete new simulations, and resulted in -19.3% to 23.7% increment compared with original simulations for super hybrid rice and inbred super rice (**Figure 5**). Among these parameters, enhancement of total canopy nitrogen concentration through improvement in SLN_ave_ (TCN-SLN_ave_) exhibited the greatest increase in AM_DAY_, and the increment averaged across the cultivars was 18.2%. We noted that the enhancement of LAI_can_ had a consistently negative impact on AM_DAY_ across the cultivars, with a maximal reduction of 19.3% in YLY3218. While the increment of AMDAY through *V*_cmax_ and β exhibited a slight impact on AM_DAY,_ the improvement of which was, however, too little to be the promising target for improving AM_DAY_.

Consistent with the tillering stage, *J*_max_, SLN_ave_, and the TCN-SLN_ave_ were still the promising target for improving AM_DAY_. While the increment in LAI_can_ minimizes the value of AM_DAY_ of YLY5867, which was 21.80 g m^-2^. We further found that the effect of *K*_N_ on the increment of AM_DAY_ was weakened in ZD11 and NJ9108.

### 3.5 AM_DAY_ simulated based on replaced parameters

**Figure 6:**
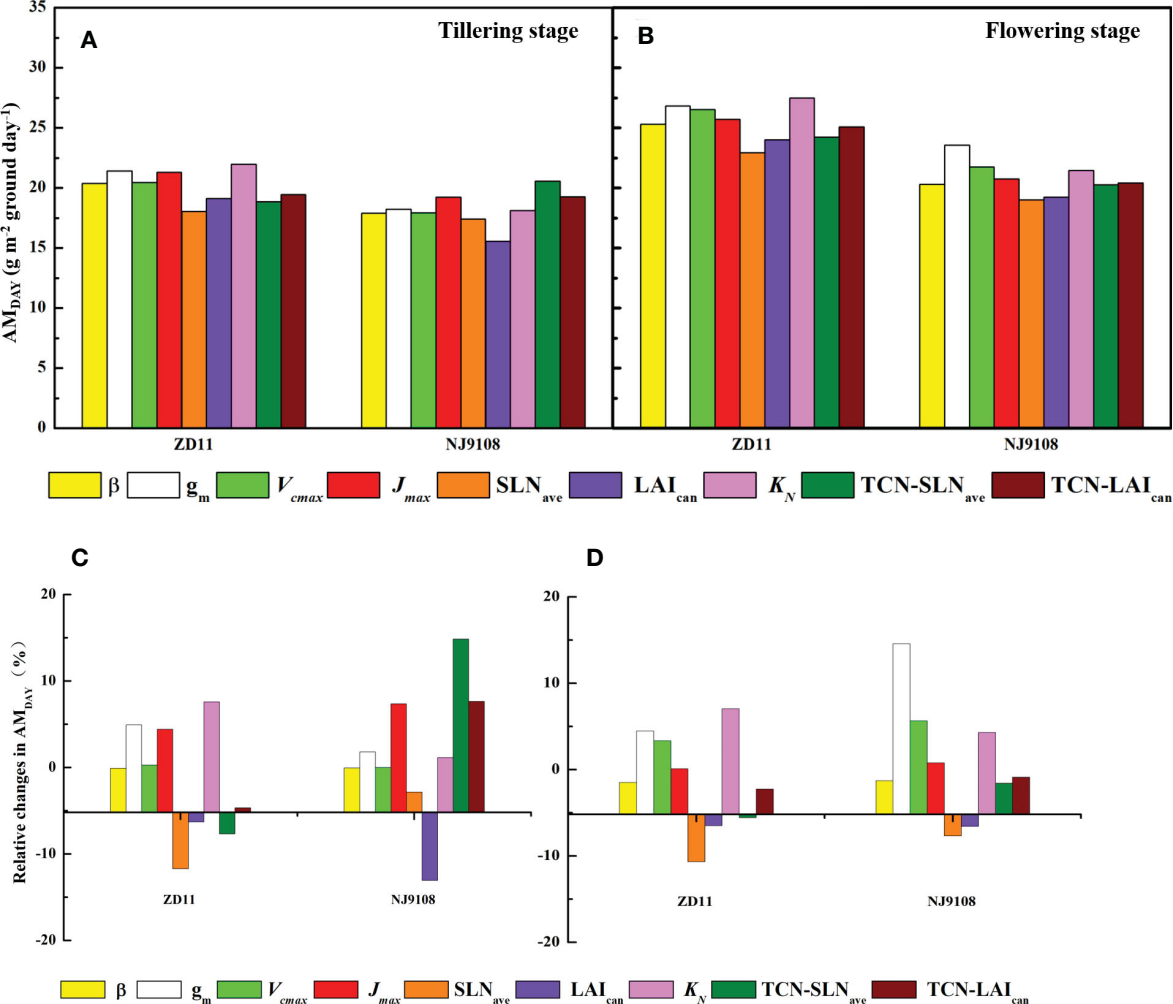
AM_DAY_ based on replaced parameters **(A, B)** and percentage change in AM_DAY_ **(C, D)** for different rice cultivars during tillering **(A, C)** and flowering stage **(B, D)**. The simulated values based on replaced parameters of YLY5867 or YLY3218 were compared with counterparts based on observed parameters. Replaced parameters include canopy average leaf inclination relative to the horizontal (β), mesophyll conductance (*g*_m_), maximum carboxylation rate (*V*_cmax_), maximum electron transfer rate (*J*_max_), average canopy nitrogen concentration per unit leaf area (SLN_ave_), canopy leaf area index (LAI_can_), canopy photosynthetic nitrogen extinction coefficient (*K*_N_), total canopy nitrogen concentration through improvement in SLN_ave_ (TNC-SLN_ave_), total canopy nitrogen concentration through improvement in LAI_can_ (TNC-LAI_can_).

To identify the key parameters that dominate AM_DAY_ variation among the super hybrid rice and inbred super rice, we employed the canopy mechanistic model method based on replaced parameters of super hybrid rice (**Figure 6**). At tillering stage, TCN-SLN_ave_ had the largest contribution (14.8%) to the AM_DAY_ increment in NJ9108. However, if the TCN-SLN_ave_ in ZD11 was replaced by that of YLY32018, the AM_DAY_ can be decreased by 7.67%. Specially, we noted that the replacement of *J*_max_ and *K*_N_ contributed to the averaged increment of the AM_DAY_ in both the ZD11 and NJ9108.

In contrast, when performing the same analysis at flowering stage, the maximal increment in AM_DAY_ was owing to the leaf *g*_m_, by 14.6% in NJ9108 and 4.47% in ZD11. The contribution of *J*_max_ to the AM_DAY_ increment was substantially weaker, while most of the variation in AM_DAY_ was due to *K*_N_, especially in ZD11, with an increment of 7.05%.

## 4 Discussion

The utilization of super rice plays an important role in high-yield production of rice over the past several decades in China. Nevertheless, there are significant differences in grain yield between the super hybrid rice and inbred super rice, i.e., YLY3218 and YLY5867 were manifested by higher grain yield (**Figures 1A**, **S3**), which was mainly caused by larger panicle in super hybrid rice (**Table S4**). Consistently, we noted that the super hybrid rice group generally accumulated more biomass at both tillering stage and flowering stage than inbred super rice group, accompanied by larger LAI (**Figures 1B**, **3A**). In most studies, improved crop biomass production was attributed to an improving photosynthetic efficiency (Zhu et al., 2012; Qu et al., 2017). Our results showed that super hybrid rice group had higher single *A*_sat_ than inbred super rice, especially at tillering stage. Further analysis showed that higher *A*_sat_ might be attributed to the synchronous improvement of CO_2_ diffusion capacity and biochemical capacity (**Tables 1**, **2**), and suggested that the nitrogen concentration per leaf area (SLN) did not contribute that much (**Figure 2B**). Similar results were also observed by Wu et al. (2019) and He et al. (2017). Moreover, *A*_sat_ in ZD11 was comparable to that of super hybrid rice at flowering stage, which indicated that the single-leaf photosynthesis advantages of the super hybrid rice cultivars were not achieved at all growth stages (**Figure 2A**). In this sense, leaf *A*_sat_ is not always a limiting factor for grain yield and crop biomass (Evans and Dunstone, 1970; Zhao et al., 2008). For example, the decreasing *A*_sat_ of super hybrid rice could be compensated by the change in LAI, resulting in an almost unimpacted yield. Additionally, by conducting the correlation analysis, the strong positive relationships between LAI and yield at both tillering and flowering stages were observed (tillering stage: *r* = 0.995, *P*< 0.01; flowering stage: *r* = 0.987, *P*< 0.05), while *A*_sat_ merely showed a weak correlation to the yield (**Table S5**). LAI is one of the two important indicators representing plant canopy structure (Laidlaw and Withers, 1998). These results were in line with the findings of Shi et al. (2019), who further stated that the synergetic changes of *A*_sat_ and LAI could contribute to higher canopy photosynthesis.

Improving canopy photosynthesis is regarded as a major target to improve crop biomass production and yield potential (Song et al., 2016). To explore the optimal factors contributing to substantial higher *A*_sat_ in super rice group at two growth stages, we performed a canopy photosynthetic model based on a 20% enhancement in each targeted parameter (**Figures 5A, B**). In this study, we examined AM_DAY_ as a surrogate of canopy photosynthetic rate. Results showed that improvement of TCN-SLN_ave_ had the greatest increment in AM_DAY_ at both tillering stage and flowering stage, with an average increase of 18.1% among cultivars at tillering stage and 10.7% at flowering stage (**Figures 5C, D**). Especially, AM_DAY_ could also be significantly elevated by increasing the averaged SLN_ave_, and a maximal increase of AM_DAY_ by 12.8% was reached in YLY3218 (**Figure 5C**). Nitrogen is one of the most important limiting resources for plant growth. Hikosaka et al. (2016) indicated that the distribution of SLN_ave_ with a leaf canopy is one of the most essential factors for canopy photosynthesis, which depended mostly on LAI (Moreau et al., 2012). In contrast, improvement of LAI presented the greatest negative effect on AM_DAY_ in both cultivars, which decreased AM_DAY_ by averaging 13.1% at tillering stage and 9.3% at flowering stage (**Figure 5D**). This is partly because of the lack of synergistic changes in leaf angle and nitrogen distribution. Especially when the plant nitrogen content is not sufficient, the gain of increasing LAI for total canopy photosynthesis was compensated by the decrease of *A*_sat_, which even led to a decrease in AM_DAY_. This is again supported by the result that a together improvement of LAI and canopy nitrogen accumulation (TCN-LAI_can_) can increase AM_DAY_ by up to 9.33%. As reported by previous studies, for plants with a higher LAI, the further increase in plant canopy photosynthesis depends on a more vertical leaf angle at the top of the canopy and a more optimal nitrogen distribution within the canopy (Zhu et al., 2010; Long et al., 2016).

Notably, the improvement of *J*_max_ can also be used as a potential route to further improve super rice, especially for super hybrid rice; while the increase in *V_c_*_max_ had rarely affected AM_DAY_ enhancement (**Figures 5C, D**), indicating the assimilate rate of leaf fractions were electron transport limited, which was in agreement with Wu et al. (2018). In the present canopy model, 20% elevation of *J*_max_ resulted in a 5.78% increase in AM_DAY_. In well-fertilized C3 crops, controls on photosynthetic capacity should be shared between *V*_cmax_ and *J*_max_ (Zhu et al., 2010). This is further supported by previous studies that overexpression of the RiesKe Fes protein in Arabidopsis led to increased photosynthesis, biomass, and grain yield, by enhancing the rate of electron transport (Yamori et al., 2016; Simkin et al., 2017). However, Sinclair et al. (2004) assessed the impact of a 50% increase in the production of mRNA for the synthesis of Rubisco, only a 6% increase or even a 6% decrease was observed in yield, which depended on whether there is extra nitrogen accumulation. The foregoing has established that at the current atmospheric CO_2_ concentration, generation of RuBP during tillering stage was important for the increase of canopy photosynthesis of super rice cultivars.

In the present study, we observed a higher AM_DAY_ in super hybrid rice group at tillering stage, while ZD11 had a comparable AM_DAY_ compared with super hybrid rice at flowering stage (**Figure 4**). As a composition of canopy structure, erect leaves are undoubtedly the most conducive to the enhancement of canopy photosynthetic efficiency and AM_DAY_. A canopy with a gradually increased β can increase the daily integral of carbon uptake by as much as 40%, compared to a canopy with horizontal leaves (Long et al., 2016). In the present study, however, β value only showed slight differences between cultivars (**Figure 3B**). For rice, one possibility could be that the canopy architecture had been effectively optimized for maximum light capture through breeding (Horton, 2000). In this sense, what are the major factors controlling the different canopy photosynthesis between super hybrid rice and inbred super rice? To answer this question, we performed single-factor substitution analysis, i.e., based on the superior parameters of the two super hybrid rice cultivars, the corresponding values were substituted for the two inbred super rice cultivars, one by one, and so on, at tillering and flowering stages, respectively. Results showed that for high AM_DAY_ in super hybrid rice, *g*_m_, *K*_N_, and *J*_max_ were predicted to have the largest contributions (**Figures 6A, B**). This is supported by a previous study that rice productivity could be significantly improved by mining the parameters determining light-limited photosynthesis (Horton, 2000). According to Zhu et al. (2022), a higher ratio of the extinction coefficient for effective leaf nitrogen to the light extinction coefficient generally led to enhanced canopy photosynthesis and dry matter content. At flowering stage, the improvement of super inbred rice was attributed to its higher SLN_ave_. Previous studies showed that the leaf nitrogen concentration was the key factor in determining photosynthetic capacity on a scale of single leaf or canopy (Schnier et al., 1990; Pao et al., 2019). Crop nutrient utilization and photosynthetic nitrogen use efficiency at late development stage were the preconditions for maintaining canopy photosynthetic advantage (Shi et al., 2019).

In summary, the improvement of canopy photosynthesis is considered to be one of the major goals to produce super high yield in the new era, in which the choice of superior traits is the most important approach. Our model showed that keeping a higher LAI and *A*_sat_ during tillering stage is of great importance for the canopy photosynthesis increment of super rice, which needs to be supported by the optimal canopy nitrogen content. For ZD11 and NJ9108, the improvement of *J*_max_, *g*_m_, and *K*_N_ had the largest impact on canopy photosynthesis compared with YLY3218 and YLY5867. These identified parameters can be used as useful targets to further improve rice yield of the tested rice varieties. It was foreseeable that the system method and analysis allowed us to suggest strategies to further improve the productivity of these superior rice varieties. The methods presented here can also be used to identify the key targets attribute to the increase in other rice lines.

## Data availability statement

The raw data supporting the conclusions of this article will be made available by the authors, without undue reservation.

## Author contributions

YP: Conceptualization, Investigation, Methodology, Software, Validation, Writing - review and editing; YCa: Investigation, Methodology, Formal analysis, Writing - original draft; YCh: Investigation, Software; XM: Writing - review and editing; GW: Supervision, Funding acquisition, Resources; MW and SG: Conceptualization, Supervision, Funding acquisition, Resources, Writing - review and editing. All authors contributed to the article and approved the submitted version.
