# Supplementary material for: Identification of photosynthetic parameters for superior yield of two super hybrid rice varieties: A cross-scale study from leaf to canopy
Source: Front Plant Sci. 2023 Feb 14;14:1110257. doi: 10.3389/fpls.2023.1110257 (PMC9971572; doi:10.3389/fpls.2023.1110257)
Supplement: Supplementary file 1 [file DataSheet_1.docx]

**Supplementary data**

**Title:** **Identification of photosynthetic parameters for superior yield of two super hybrid rice varieties: A cross-scale study from leaf to canopy**

**Authors:** Yonghui Pan†, Yiwen Cao†, Yixiao Chai, Xusheng Meng, Guanjun Huang*, Shiwei Guo*

**TABLE S1.** Description of parameters and their values or equations in the Diurnal Canopy Photosynthesis Simulator (DCaPS).

**TABLE S2.** Temperature response parameters in photosynthetic model.

**TABLE S3.** Parameters specific for rice in the present study used for simulating daily canopy photosynthesis.

**TABLE S4.** Effects of N rates on yield components of different rice varieties.

**TABLE S5.** Correlation analysis between net photosynthesis rate under saturated light (*A*_sat_), leaf area index (LAI) and yield.

**FIGURE S1** Daily mean temperature, precipitation and photosynthetic active radiation (PAR) during rice growing season at Rugao County, Jiangsu Province, China in 2020.

**FIGURE S2** Schematic of the Diurnal canopy photosynthesis simulator (DCaPS, recomposed from Wu et al. (2018).

**FIGURE S3** Effects of N rates on yield at maturity of different rice varieties.

**TABLE S1. Description of parameters and their values or equations in the Diurnal Canopy Photosynthesis Simulator (DCaPS)**

| **Symbol** | **Description** | **Units** | **Value or Equation** |
| --- | --- | --- | --- |
| **Environment parameters** | | | |
| S_o_ | Total daily extra-terrestrial solar radiation†^(1)^ | MJ m^-2^ ground day^-1^ | $\frac{24}{\pi}*\frac{\mathrm{sch}}{{Rl}^{2}}*(Wl^{\circ}*\frac{\pi}{180}*sinLat*sinDl+\sin Wl^{\circ}*\mathrm{cosLat}*\cos Dl)$ |
| S_g_ | Total daily incident solar radiation†^(2)^ | MJ m^-2^ ground day^-1^ | $S_{o}*RATIO$ |
| RATIO | Atmospheric transmission ratio†^(2)^ | dimensionless | 0.75 |
| sch | Solar constant in energy unit per hour†^(2)^ | J m^-2^ ground hr^-1^ | 4896000 |
| sc | Solar constant in energy unit per second†^(2)^ | J m^-2^ ground s^-1^ | 1360 |
| Lat | Latitude in radians | radians | Table S3 |
| Rl | Radius vector†^(1)^ | radians | 1/$\sqrt{0.033*\cos\left( 360*\frac{\mathrm{Day}}{365} \right)}$ |
| *Dl* | Solar declination†^(1)^ | radians | $23.45*\sin(2*\pi*\frac{248+Day}{365})*\frac{\pi}{180}$ |
| Wl° | Sunset hour-angle†^(1)^ | ° | $\cos^{-1}[\left( -\tan\mathrm{Lat}*\tan Dl \right)*\frac{180}{\pi}]$ |
| *Ll* | Day length†^(1)^ | hr | $Wl^{\circ}/7.5$ |
| Day | Day of year | dimensionless | Table S3 |
| VPD_a_ | Air vapour pressure deficit†^(3)^ | kPa | $\mathrm{SVP}_{a}-\mathrm{SVP}_{d}$ |
| SVP_a_ | Air saturated vapour pressure†^(3)^ | kPa | $610.7*exp(17.4*\frac{T_{a}}{239+T_{a}})/1000$ |
| SVP_d_ | Air dew-point vapour pressure†^(3)^ | kPa | $610.7*exp(17.4*\frac{T_{\min}}{239+T_{\min}})/1000$ |
| C_a_ | Air CO_2_ partial pressure | μbar | 405.3 |
| O_a_ | Air O_2_ partial pressure | μbar | 210000 |
| I_o_ | Total incident solar radiation†^(4)^ | MJ m^-2^ ground s^-1^ | $S_{g}*\pi*sin (\pi*t_{\mathrm{frac}})/(2*Ll*3600)$ |
| I_dir_ | Incident direct radiation†^(2)^ | MJ m^-2^ ground s^-1^ | $I_{o}-I_{\mathrm{dif}}$ |
| I_dif_ | Incident diffuse radiation†^(2), (5)^ | MJ m^-2^ ground s^-1^ | $0.17*sc*\sin\left( \alpha_{sun} \right)/1000000$ |
| PAR_o_ | Total incident photosynthetic active radiation | μmol PAR m^-2^ ground^-1^ | $\mathrm{PAR}_{\mathrm{dir}}+\mathrm{PAR}_{\mathrm{dif}}$ |
| PAR_dir_ | Direct incident photosynthetic active radiation†^(6)^ | μmol PAR m^-2^ ground^-1^ | $I_{\mathrm{dir}}*0.5*4.56*1000000$ |
| PAR_dif_ | Diffuse incident photosynthetic active radiation†^(6)^ | μmol PAR m^-2^ ground^-1^ | $I_{\mathrm{dif}}*0.5*4.25*1000000$ |
| PAR_abs,can_ | Absorbed PAR of canopy†^(7), (8)^ | μmol PAR m^-2^ ground^-1^ | $\left( 1-\rho_{cb} \right)*\mathrm{PAR}_{\mathrm{dir}}*\left[ 1-\exp\left( -K_{b}*\mathrm{LAI}_{\mathrm{can}} \right) \right]+\left( 1-\rho_{cd} \right)*\mathrm{PAR}_{\mathrm{dif}}*[1-\exp\left( -K_{d}*\mathrm{LAI}_{\mathrm{can}} \right)]$ |
| PAR_abs,sun_ | PAR absorbed by the sunlit leaves†^(7), (8)^ | μmol PAR m^-2^ ground^-1^ | $\left( 1-\sigma\right)*I_{dir}*\left[ 1-\exp\left( -K_{b}*\mathrm{LAI}_{\mathrm{can}} \right) \right]+\left( 1-\rho_{cd} \right)*I_{\mathrm{dif}}*\left[ 1-\exp\left( -K_{d}+k_{b} \right)*\mathrm{LAI}_{\mathrm{can}} \right]*\frac{K_{d}}{K_{d}+k_{b}}+I_{\mathrm{dir}}*\{\left( 1-\rho_{cb} \right)*\left[ 1-\exp\left( -K_{b}+k_{b} \right)*\mathrm{LAI}_{\mathrm{can}} \right]*\frac{K_{b}}{K_{b}+k_{b}}-\left( 1-\sigma\right)*\left[ 1-\exp\left( -{2*K}_{b}*\mathrm{LAI}_{\mathrm{can}} \right) \right]*0.5\}$ |
| PAR_abs,sh_ | PAR absorbed by the shaded leaves | μmol PAR m^-2^ ground^-1^ | $\mathrm{PAR}_{abs,can}-\mathrm{PAR}_{abs,sun}$ |
| t_frac_ | t as a fraction of *Ll* passed at t from t_sunrise_ | dimensionless | $\left[ t-\left( 12-0.5*Ll \right) \right]/Ll$ |
| t_sunrise_ | Time of sunrise | hr | $12-0.5*Ll$ |
| t_sunset_ | Time of sunset | hr | $12+0.5*Ll$ |
| *α_sun_* | Angle of solar elevation | radians | $\sin^{-1} \{sinLat*sinDl+cosLat*cosDl*\cos\left[ Ll*\left( t_{\mathrm{frac}}-0.5 \right)*\frac{\pi}{12} \right]\}$ |
| T_a_ | Air temperature†^(9)^ | ℃ | $\left\{ \begin{aligned} \left( T_{\max}-T_{\min} \right)*\sin\left( \frac{\pi*m}{Ll+2*x_{\mathrm{lag}}} \right)+T_{\min}, for t_{\mathrm{Tmin}} &\leq t<t_{\mathrm{sunset}} \\ T_{\min}+\left( T_{\mathrm{sunset}}-T_{\min} \right)*\exp\left( -\frac{n*y_{\mathrm{lag}}}{24-Ll} \right), for t_{\mathrm{sunset}}\leq t< t_{\mathrm{Tmin}} \end{aligned} \right.$ |
| T_max_ | Maximum T_a_ of DAY | ℃ | Table S3 |
| T_min_ | Minimum T_a_ of DAY | ℃ | Table S3 |
| m | Amount of time since time of minimum temperature†^(9)^ | hr | $\left\{ \begin{aligned} \left( T_{\max}-T_{\min} \right)*\sin\left( \frac{\pi*m}{Ll+2*x_{\mathrm{lag}}} \right)+T_{\min}, for t_{\mathrm{Tmin}} &\leq t<t_{\mathrm{sunset}} \\ T_{\min}+\left( T_{\mathrm{sunset}}-T_{\min} \right)*\exp\left( -\frac{n*y_{\mathrm{lag}}}{24-Ll} \right), for t_{\mathrm{sunset}}\leq t< t_{\mathrm{Tmin}} \end{aligned} \right.$ |
| n | Amount of time since t_sunset_†^(9)^ | hr | $\left\{ \begin{aligned} \left( T_{\max}-T_{\min} \right)*\sin\left( \frac{\pi*m}{Ll+2*x_{\mathrm{lag}}} \right)+T_{\min}, for t_{\mathrm{Tmin}} &\leq t<t_{\mathrm{sunset}} \\ T_{\min}+\left( T_{\mathrm{sunset}}-T_{\min} \right)*\exp\left( -\frac{n*y_{\mathrm{lag}}}{24-Ll} \right), for t_{\mathrm{sunset}}\leq t< t_{\mathrm{Tmin}} \end{aligned} \right.$ |
| x_lag_ | Lag coefficient for the maximum temperature from t_sunrise_†^(9)^ | dimensionless | 1.8 |
| y_lag_ | Lag coefficient for the night-time temperature from t_sunrise_†^(9)^ | dimensionless | 2.2 |
| z_lag_ | Lag coefficient for the minimum temperature from t_sunrise_†^(9)^ | dimensionless | 1 |
| **Canopy architecture parameters** | | | |
| LAI_can_ | Canopy leaf area index | m^2^ leaf m^-2^ ground | Table S3 |
| LAI_sun_ | LAI of sunlit leaf fraction†^(8)^ | m^2^ leaf m^-2^ ground | $\left[ 1-\exp\left( -k_{b}*\mathrm{LAI}_{\mathrm{can}} \right) \right]/k_{b}$ |
| LAI_sh_ | LAI of shade leaf fraction†^(8)^ | m^2^ leaf m^-2^ ground | $\mathrm{LAI}_{\mathrm{can}}-\mathrm{LAI}_{\mathrm{sun}}$ |
| L | Cumulative LAI from the top of the canopy | m^2^ leaf m^-2^ ground^-1^ | Table 3 |
| K_b_ | Direct and scattered direct PAR extinction coefficient†^(7), (8)^ | dimensionless | $k_{b}*\sqrt{1-\sigma}$ |
| K_d_ | Diffuse and scattered diffuse PAR extinction coefficient†^(7), (8)^ | dimensionless | $k_{d}*\sqrt{1-\sigma}$ |
| k_b_ | Direct radiation extinction coefficient†^(7), (8), (10)^ | dimensionless | $G/sin\alpha$ |
| k_d_ | Diffuse PAR extinction coefficient†^(8)^ | dimensionless | 0.78 |
| σ | Leaf level scattering coefficient for PAR† ^(8)^ | dimensionless | 0.15 |
| *ρ_cb_* | Canopy level reflection coefficient for direct PAR† ^(8)^ | dimensionless | $1-exp(2*\rho_{h}*\frac{k_{b}}{1+k_{b}})$ |
| *ρ_h_* | Reflection coefficient of a canopy with horizontal leaves† ^(8)^ | dimensionless | $\frac{1-\sqrt{(1-\sigma)}}{1+\sqrt{(1-\sigma)}}$ |
| *ρ_cd_* | Canopy level reflection coefficient for diffuse PAR† ^(8)^ | dimensionless | 0.036 |
| G | Leaf shadow projection coefficient† ^(10)^ | dimensionless | $\left\{ \begin{aligned} \cos\alpha*\sin\beta, for \alpha&\leq\beta\\ \frac{2}{\pi}*sin\alpha*cos\beta*sin\omega+\left( 1-\frac{\omega^{\circ}}{90} \right)*\cos\alpha*\sin\beta, for \alpha>\beta\end{aligned} \right.$ |
| ω | Parameter used for calculation of G† ^(10)^ | ° | $\cos^{-1} (cot\alpha*sin\beta)$ |
| β | Canopy average leaf inclination relative to the horizontal† ^(8)^ | radians | 60° |
| T_l_ | Leaf temperature | ℃ | T_a_ |
| **Canopy nitrogen status parameters** | | | |
| SLN_ave_ | Average canopy nitrogen concentration per unit leaf area | g N m^-2^ leaf | Table S3 |
| SLN_ratio_top_ | Ratio of SLN_ave_ to SLN_o_ | dimensionless | Table S3 |
| SLN_top_ | SLN at the top of the canopy | g N m^-2^ leaf | Table S3, $\mathrm{SLN}_{ratio\_top}*\mathrm{SLN}_{\mathrm{ave}}$ |
| SLN_L_ | SLN at L† ^(8)^ | g N m^-2^ leaf | $\left( \mathrm{SLN}_{\mathrm{top}}-\mathrm{SLN}_{b} \right)*\exp\left( -\mathrm{SLN}_{b}*\frac{L}{\mathrm{LAI}_{\mathrm{can}}} \right)+\mathrm{SLN}_{b}$ |
| N_ave_ | Average canopy nitrogen concentration per unit leaf area | mmol N m^-2^ leaf | $\mathrm{SLN}_{\mathrm{ave}}*1000/14$ |
| N_top_ | SLN at the top of the canopy | mmol N m^-2^ leaf | $\mathrm{SLN}_{\mathrm{top}}*1000/14$ |
| N_L_ | SLN at L | mmol N m^-2^ leaf | $\mathrm{SLN}_{L}*1000/14$ |
| N_b_ | SLN when leaf photosynthesis = 0† ^(11)^ | mmol N m^-2^ leaf | 21.43 |
| TNC | Total canopy nitrogen concentration† ^(8)^ | mmol N m^-2^ ground | $\mathrm{LAI}_{\mathrm{can}}*\{\left( N_{\mathrm{top}}-N_{b} \right)*[1-\exp\left( -K_{N} \right)]/K_{N}+N_{b}$ |
| *K_N_* | Canopy photosynthetic nitrogen extinction coefficient | dimensionless | $-2*ln\frac{N_{\mathrm{ave}}-N_{b}}{N_{\mathrm{top}}-N_{b}}$ |
| **Photosynthetic parameters** | | | |
| χ_v_ | Slope of linear relationship between *V_cmax_* per leaf area at 25 ℃ and N†^(8), (12), (13)^ | μmol CO_2_ mmol^-1^ N s^-1^ | Table S3, $\frac{V_{cmax25}}{N_{\mathrm{ave}}-N_{b}}$ |
| χ*_J_* | Slope of linear relationship between *J_max_* per leaf area at 25 ℃ and N†^(8), (12), (13)^ | μmol CO_2_ mmol^-1^ N s^-1^ | Table S3, $\frac{J_{max25}}{N_{\mathrm{ave}}-N_{b}}$ |
| χ_R_ | Slope of linear relationship between R_d_ per leaf area at 25 ℃ and N†^(8), (12), (13)^ | μmol CO_2_ mmol^-1^ N s^-1^ | 0.01χ_v_ |
| χ_P_ | Values of χ_v_,χ*_J_* or χ_R_ | μmol CO_2_ mmol^-1^ N s^-1^ | χ_v_,χ*_J_* or χ_R_ |
| P_can25_ | Values of *V_cmax_*, *J_max_* or R_d_ for the whole canopy at 25 ℃†^(8)^ | μmol CO_2_ m^-2^ ground^-1^ s^-1^ | $\mathrm{LAI}_{\mathrm{can}}*\chi_{P}*\left( N_{\mathrm{top}}-N_{b} \right)*\frac{1-exp(-K_{N})}{K_{N}}$ |
| P_sun25_ | Values of *V_cmax_*, *J_max_* or R_d_ for the sunlit leaf fraction at 25 ℃†^(8)^ | μmol CO_2_ m^-2^ ground^-1^ s^-1^ | $\mathrm{LAI}_{\mathrm{can}}*\chi_{P}*\left( N_{\mathrm{top}}-N_{b} \right)*\frac{1-exp(-K_{N}-k_{b}*\mathrm{LAI}_{\mathrm{can}})}{K_{N}+k_{b}*\mathrm{LAI}_{\mathrm{can}}}$ |
| P_sh25_ | Values of *V_cmax_*, *J_max_* or R_d_ for the shade leaf fraction at 25 ℃ | μmol CO_2_ m^-2^ ground^-1^ s^-1^ | $P_{can25}-P_{sun25}$ |
| *V_camx_g25_* | Maximum Rubisco carboxylation rate per ground area at 25 ℃ | μmol CO_2_ m^-2^ ground^-1^ s^-1^ | Table S2 |
| *J_max_g25_* | Maximum rate of electron transport per ground area at 25 ℃ | μmol CO_2_ m^-2^ ground^-1^ s^-1^ | Table S2 |
| R_d_g25_ | Leaf day respiration per ground area at 25 ℃ | μmol CO_2_ m^-2^ ground^-1^ s^-1^ | Table S2 |
| *K_c_* | Michaelis constant of Rubisco for CO_2_ | μbar | Table S2 |
| *K_o_* | Michaelis constant of Rubisco for O_2_ | μbar | Table S2 |
| *A* | Net CO_2_ assimilation rate†^(14)^ | μmol CO_2_ m^-2^ ground^-1^ s^-1^ | $min\{A_{c,\varepsilon},A_{j,\varepsilon}\}$ |
| *A_c_* | Rubisco limited net CO_2_ assimilation rate†^(14)^ | μmol CO_2_ m^-2^ ground^-1^ s^-1^ | $\left( C_{C}-\Gamma^{*} \right)*\frac{V_{cmax}}{\left[ C_{C}+K_{C}*\left( 1+\frac{O_{C}}{K_{O}} \right) \right]}-R_{d}$ |
| *A_j_* | Electron transport limited net CO_2_ assimilation rate†^(14)^ | μmol CO_2_ m^-2^ ground^-1^ s^-1^ | $\left( C_{C}-\Gamma^{*} \right)*\frac{J}{4*C_{C}+8*\Gamma^{*}}-R_{d}$ |
| *Γ** | CO_2_ compensation point in the absence of R_d_†^(14)^ | μbar | $\Upsilon^{*}*O_{C}$ |
| *γ** | Half the reciprocal of *S_c/o_*†^(14)^ | dimensionless | $0.5/S_{C/O}$ |
| *S_c/o_* | Relative CO_2_/O_2_ specificity of Rubisco†^(14)^ | bar bar^-1^ | $\frac{K_{O}}{K_{C}}*\frac{V_{cmax}}{V_{omax}}$ |
| *V_camx_/V_oamx_* | Ratio of maximum Rubisco carboxylation rate to maximum Rubisco oxygenation rate | dimensionless | Table S2 |
| *J* | Potential electron transport rate†^(14)^ | μmol e- m^-2^ ground s^-1^ | $\frac{I_{2,\varepsilon}+J_{max,\varepsilon}-\sqrt{\left( I_{2,\varepsilon}+J_{max,\varepsilon} \right)^{2}-4*\theta*J_{max,\varepsilon}*I_{2,\varepsilon}}}{2*\theta}$ |
| *θ* | Empirical curvature factor†^(14)^ | dimensionless | 0.7 |
| *f* | Spectral correction factor†^(14)^ | dimensionless | 0.15 |
| *I_2_* | PAR absorbed by Photosystem II†^(14)^ | μmol PAR m^-2^ ground^-1^ s^-1^ | $\mathrm{PAR}_{abs,\varepsilon}*(1-f)/2$ |
| **CO_2_ diffusion parameters** | | | |
| C_i_ | Intercellular airspace CO_2_ partial pressure | μbar | Table 3，$\frac{C_{i}}{C_{a}}*C_{a}$ |
| C_c_ | Chloroplast CO_2_ partial pressure†^(15)^ | μbar | $\frac{C_{i}}{C_{a}}*C_{a}-\frac{A_{\varepsilon}}{g_{m,\varepsilon}}$ |
| O_l_ | O_2_ partial pressure inside leaves | μbar | O_a_ |
| O_c_ | O_2_ partial pressure at chloroplast | μbar | O_l_ |
| a | Slope of linear relationship between C_i_/C_a_ and VPD_a_†^(16)^ | kPa^-1^ | -0.12 |
| b | Intercept of linear relationship between C_i_/C_a_ and VPD_a_†^(16)^ | dimensionless | 0.9 |
| C_i_/C_a_ | Ratio of C_i_ to C_a_ | dimensionless | Table S3 |
| g_m_ | Mesophyll conductance per leaf area | mol CO_2_ m^-2^ leaf s^-1^ bar^-1^ | Table S3 |
| g_m_g_ | Mesophyll conductance per ground area | mol CO_2_ m^-2^ ground s^-1^ bar^-1^ | $g_{m}*\mathrm{LAI}_{\varepsilon}$ |
| **Final output** | | | |
| *A*_can,DAY_ | Daily canopy photosynthesis | μmol CO_2_ m^-2^ ground day^-1^ | $\sum_{i=[t_{\mathrm{sunrise}}]}^{[t_{\mathrm{sunset}}]} (A_{can,inst,i}*3600)$ |

Parameters with subscript ε means it is determined separately for sunlit leaf fraction and shade leaf fraction; Values of parameters that listed in Table S3 are specific for rice in the present study; Reference†^(1)-(16)^: Brock, 1981; Hammer and Wright, 1994; Goudriaan and van Laar, 1994; Charles-Edwards, 1986; Collares-Pereira and Rabl, 1979; Monteith and Unsworth, 2013; Leuning et al., 1995; de Pury and Farquhar, 1997; Parton and Logan, 1981; Ducan et al., 1967; Yin and van Laar, 2005; Evans, 1983; Harley et al., 1992; Farquhar et al., 1980; Wong et al., 1979; Zhang and Nobel, 1996.

**TABLE S2 Temperature response parameters in photosynthetic model.**

| **Parameter** | **Units** | **P_25_** | **c (dimensionless)** | **b (K)** | **Equation** | **Reference** |
| --- | --- | --- | --- | --- | --- | --- |
| *K_c_* | μbar | 272.4 | 32.7 | 9741.4 | $P=P_{25}*exp(c-\frac{b}{T_{l}+273})$ | Bernacchi et al., 2002 |
| *K_o_* | μbar | 165800 | 9.6 | 2853.0 |  |  |
| *V_cmax_/V_omax_* | - | 4.6 | 13.2 | 3945.7 |  |  |
| *V_cmax_* | μmol CO_2_ m^-2^ s^-1^ | Variable | 26.4 | 7857.8 |  | Bernacchi et al., 2001 |
| R_d_ | μmol CO_2_ m^-2^ s^-1^ | Variable | 18.7 | 5579.7 |  |  |
|  | | **P_25_** | T_opt_ (℃) | Ω **(K)** | $P=P_{25}*exp[\left( \frac{25-T_{\mathrm{opt}}}{\Omega} \right)^{2}- \left( \frac{T_{l}-T_{\mathrm{opt}}}{\Omega} \right)^{2}$] |  |
| *J_max_* | μmol CO_2_ m^-2^ s^-1^ | Variable | 28.8 | 15.5 |  | Farquhar et al., 1980 |
| g_m_ | mol CO_2_ m^-2^ ground^-1^ s^-1^ bar^-1^ | Table 3 | 34.3 | 20.8 |  | Bernacchi et al., 2002 |

P_25_, value of modeled parameter at 25 ℃; P, value of modeled parameter at T_l_; T_opt_ and T_l_, optimum and leaf temperature used in equations.

**TABLE S3 Parameters specific for rice in the present study used for simulating daily canopy photosynthesis.**

| **Parameter** | **units** | **Value** | |
| --- | --- | --- | --- |
|  |  | **Tillering stage** | **Flowering stage** |
| **Environment parameters** | | | |
| Lat | radians | 32.23 | |
| Day | dimensionless | 203 | 245 |
| T_max_ | ℃ | 34.6 | 31.3 |
| T_min_ | ℃ | 27.8 | 21.7 |
| **Canopy nitrogen status parameters** | | | |
| LAI_can_ | m^2^ leaf m^-2^ ground | 3.85, 3.84, 3.26, 3.17 | 5.09, 5.06, 4.43, 4.19 |
| β | ° | 60.3, 61.3, 62.1, 63.0 | 54.0, 59.1, 59.3, 54.0 |
| **Canopy nitrogen status parameters** | | | |
| SLN_ave_ | g N m^-2^ leaf | 0.52, 0.57, 0.75, 0.60 | 0.75, 0.63, 0.93, 0.93 |
| SLN_ratio_top_ | dimensionless | 0.54, 0.78, 0.97, 0.78 | 0.78, 0.79, 0.94, 0.89 |
| SLN_top_ | g N m^-2^ leaf | 0.96, 0.73, 0.77, 0.77 | 0.96, 0.80, 0.99, 1.04 |
| **Photosynthetic parameters** | | | |
| χ_v_ | μmol CO_2_ mmol^-1^ N s^-1^ | 2.97, 4.70, 2.72, 2.71 | 2.99, 3.45, 2.31, 1.80 |
| χ*_J_* | μmol CO_2_ mmol^-1^ N s^-1^ | 4.54, 5.72, 4.69, 4.57 | 4.70, 5.43, 4.41, 3.29 |
| *C*_i_/*C*_a_ | dimensionless | 0.700, 0.732, 0.711, 0.709 | 0.679, 0.710, 0.696, 0.693 |
| *g*_m_ | mol CO_2_ m^-2^ leaf s^-1^ bar^-1^ |  |  |

Environment parameters are indentical for each treatment, and parameters other than environment parameters are specific for YLY3218, YLN5867, ZD11, and NJ9108.

**TABLE S4 Effects of N rates on yield components of different rice varieties.**

| N rate  (kg hm^-2^) | Cultivar | Panicles  (*10^4^ hm^-2^) | | Spikelets  (panicle^-1^) | | Grain filling percentage  (%) | | 1000-grain weight  (g) | |
| --- | --- | --- | --- | --- | --- | --- | --- | --- | --- |
|  |  | 2018 | 2019 | 2018 | 2019 | 2018 | 2019 | 2018 | 2019 |
| 0 | YLY3218 | 208.4^aB^ | 192.3^aC^ | 165.3^aB^ | 212.7^aB^ | 93.0^aA^ | 93.5^aA^ | 24.4^bA^ | 25.8^cAB^ |
|  | YLY5867 | 216.0^aB^ | 175.4^aB^ | 176.5^aB^ | 189.6^aB^ | 94.3^aA^ | 95.2^aA^ | 27.4^aA^ | 29.0^aA^ |
|  | ZD11 | 213.5^aB^ | 183.6^aD^ | 118.7^bA^ | 99.2^bB^ | 93.7^aA^ | 97.5^aA^ | 27.2^aA^ | 29.4^aA^ |
|  | NJ9108 | 221.7^aB^ | 187.7^aC^ | 154.2^aA^ | 120.7^bB^ | 91.4^aA^ | 97.4^aA^ | 24.4^bA^ | 27.1^bB^ |
| 90 | YLY3218 | 249.1^aAB^ | 230.8^aBC^ | 193.5^aB^ | 244.0^aA^ | 89.4^aAB^ | 93.3^bcA^ | 24.8^bcA^ | 25.5^bB^ |
|  | YLY5867 | 266.0^aA^ | 233.8^aAB^ | 182.2^aAB^ | 249.6^aA^ | 91.5^aAB^ | 92.1^cA^ | 27.9^aA^ | 28.4^aA^ |
|  | ZD11 | 236.8^aB^ | 257.4^aC^ | 137.5^bA^ | 137.3^bA^ | 93.3^aA^ | 98.0^aA^ | 25.9^bA^ | 28.0^aB^ |
|  | NJ9108 | 237.4^aB^ | 221.5^aBC^ | 168.4^abA^ | 117.8^bB^ | 92.5^aA^ | 97.1^abA^ | 24.1^cAB^ | 28.8^aA^ |
| 180 | YLY3218 | 281.4^aA^ | 245.1^bABC^ | 241.2^aA^ | 237.3^aAB^ | 89.3^bcAB^ | 94.7^abA^ | 24.8^bA^ | 27.0^cA^ |
|  | YLY5867 | 287.6^aA^ | 258.5^bA^ | 215.5^aA^ | 210.5^aB^ | 88.9^cBC^ | 92.7^bA^ | 28.5^aA^ | 28.7^abA^ |
|  | ZD11 | 254.5^aB^ | 325.1^aB^ | 138.8^bA^ | 149.1^bA^ | 94.0^abA^ | 98.0^aA^ | 25.5^bA^ | 27.6^bBC^ |
|  | NJ9108 | 272.5^aAB^ | 254.4^bAB^ | 158.6^bA^ | 126.4^bB^ | 94.4^aA^ | 96.1^abA^ | 24.0^bAB^ | 29.4^aA^ |
| 270 | YLY3218 | 252.2^bAB^ | 291.3^bAB^ | 232.0^aA^ | 254.5^aA^ | 84.4^bB^ | 93.6^aA^ | 24.7^bA^ | 26.3^bAB^ |
|  | YLY5867 | 267.5^bA^ | 255.4^bA^ | 190.7^bAB^ | 209.5^bB^ | 88.3^aABC^ | 94.3^aA^ | 28.8^aA^ | 29.6^aA^ |
|  | ZD11 | 350.2^aA^ | 353.8^aAB^ | 134.7^cA^ | 138.6^cA^ | 92.0^aA^ | 97.7^aA^ | 24.2^bAB^ | 26.6^bCD^ |
|  | NJ9108 | 257.5^bB^ | 280.0^bAB^ | 157.8^cA^ | 142.3^cAB^ | 92.8^aA^ | 94.9^aA^ | 23.5^bAB^ | 29.0^aA^ |
| 360 | YLY3218 | 269.1^bcA^ | 307.7^bA^ | 253.3^aA^ | 252.3^aA^ | 81.3^bB^ | 92.8^aA^ | 24.5^bA^ | 26.2^bAB^ |
|  | YLY5867 | 256.6^cAB^ | 266.7^bA^ | 184.0^bAB^ | 215.2^bB^ | 85.6^bC^ | 93.1^aA^ | 28.8^aA^ | 28.8^aA^ |
|  | ZD11 | 392.3^aA^ | 393.8^aA^ | 136.9^cA^ | 151.3^cA^ | 92.2^aA^ | 96.9^aA^ | 23.2^bcB^ | 25.8^bD^ |
|  | NJ9108 | 313.7^bA^ | 310.8^bA^ | 170.7^bA^ | 162.4^cA^ | 94.1^aA^ | 94.8^aA^ | 22.3^cB^ | 28.4^aA^ |
| *ANOVA* | Variety (V) | ** | ** | ** | ** | ** | ** | ** | ** |
|  | Nitrogen (N) | ** | ** | ** | ** | ** | ns | ** | ** |
|  | V×N | ** | * | ** | ** | ** | ns | ** | ** |

Note: Different lowercases of the same N rates means significant differences among different rice varieties at the 5% level, different uppercases of the same variety means significant differences among different N rates at the 5% level. The significance test (*P* values) of the analysis of variance two-way (*ANOVA*) between V and N and their interaction (V×N) are given (** *P* <0.01; * *P* <0.05; ns, no significant difference).

**TABLE S5 Correlation analysis between net photosynthesis rate under saturated light (*A*_sat_), leaf area index (LAI) and yield.**

| **correlation coefficient *r*** | ***A*_sat_** | | **LAI** | |
| --- | --- | --- | --- | --- |
|  | **Tillering stage** | **Flowering stage** | **Tillering stage** | **Flowering stage** |
| Yield | 0.907ns | 0.681ns | 0.995** | 0.987* |

** P<0.01; *P<0.05; ns, no significant difference.


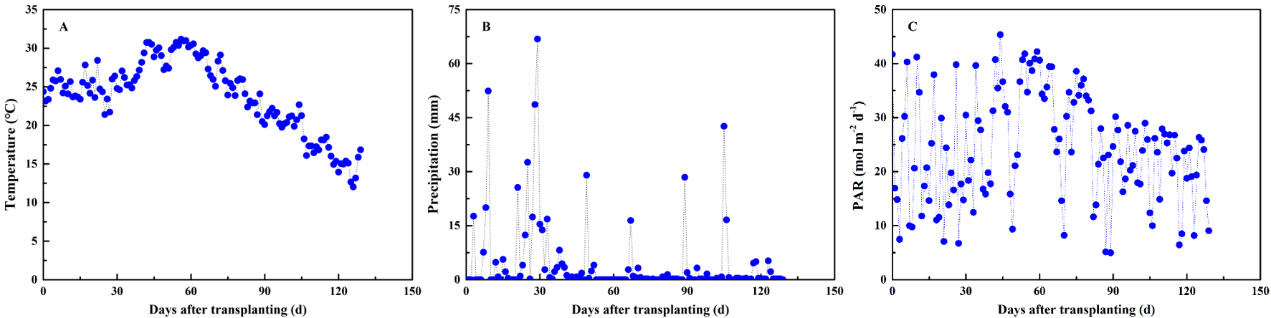


**FIGURE S1** Daily mean temerature, precipitation and photosynthetic active radiation (PAR) during rice growing season at Rugao County, Jiangsu Province, China in 2020.


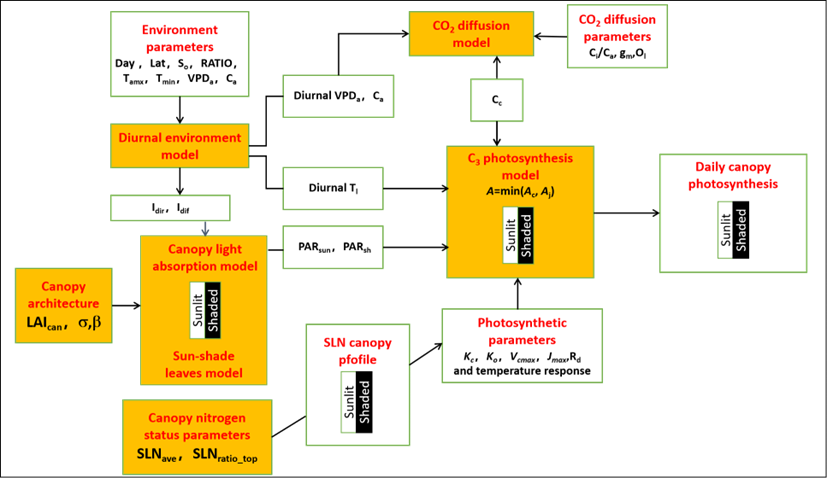


**FIGURE S2** Schematic of the Diurnal canopy photosynthesis simulator (DCaPS, recomposed from Wu et al. ( 2018). Model inputs are composed of environment, canopy architecture, canopy nitrogen status, CO_2_ diffusion, photosynthetic and temperature response parameters. Model outputs are diurnal environment variables, diurnal canopy photosynthesis. The chloroplastic CO_2_ partial pressure (C_c_) and photosynthesis are coupled and solved simultaneously. According to the combined photosynthetic model, *A_c_* and *A_j_* are solved for both sunlit leaf fraction and shaded leaf fraction by the equation:

$A_{c}or A_{j}=0.5*\{x_{1}-R_{d}+g_{m}*\left( C_{i}+x_{2} \right)-\left[ x_{1}-R_{d}+g_{m}*\left( C_{i}+x_{2} \right) \right]^{2}-4*g_{m}*[\left( C_{i}-\Gamma^{*} \right)*x_{1}-R_{d}*\left( C_{i}+x_{2} \right)$]

For *A_c_*, x_1_=*V_camx_* and x_2_=*K_c_*/(1+*K_o_*); for *A_j_*, x_1_=*J*/4 and x_2_=2*Γ^*^. When calculation of *A_c_* and *A_j_*, C_c_ is solved by equation listed in Table S1. Abbreviations: RATIO, atmospheric transmission ratio for incident solar radiation; VPD_a_, air vapour pressure deficit; PAR, photosynthetic active radiation; SLN_ave_, average canopy nitrogen concentration per unit leaf area; SLN_ratio_top_, ratio of SLN at the top of canopy to SLN_ave_; T_l_, leaf temperature; g_m_, mesophyll conductance for CO_2_; *O*_l_, O_2_ partial pressure inside leaves. Comprehensive lists of the photosynthetic parameters are given in Tables S1, S2 and S3.


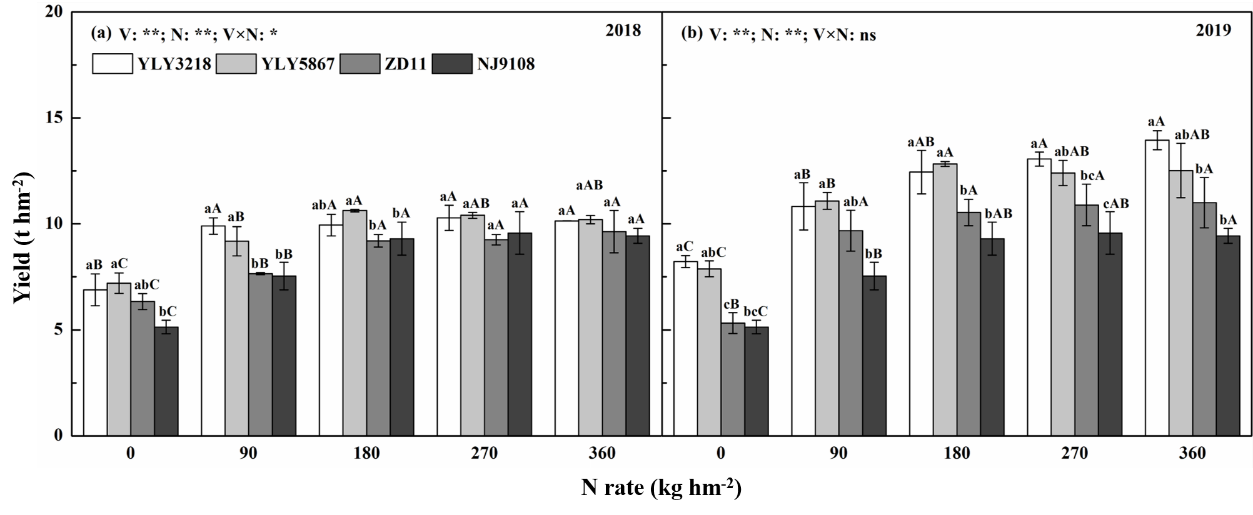


**FIGURE S3** Effects of N rates on yield at maturity of different rice varieties

Different lowercases of the same N rates means significant differences among different rice varieties at the 5% level, different uppercases of the same variety means significant differences among different N rates at the 5% level. The significance test (P values) of the analysis of variance two-way (*ANOVA*) between V and N and their interaction (V×N) are given (** P <0.01; * P <0.05; ns, no significant difference).

**References**

1. Wu, A, Doherty, A, Farquhar, G.D., Hammer, G.L., Simulating daily field crop canopy photosynthesis: an integrated sofware package. Func. Plant Biol. 45(2018) 362-377.
2. Bernacchi, C.J., Portis, A.R., Nakano, H., von Caemmerer, S, Long, S.P., Temperature response of mesophyll conductance. Implications for the determination of Rubisco enzyme kinetics and for limitations to photosynthesis in vivo. Plant Physiol. 130 (2002)1992-1998.
   3. Bernacchi, C.J, Singsaas, E.L, Pimentel, C, Portis Jr, A.R, Long, S.P., Improved temperature response functions for models of Rubisco-limited photosynthesis. Plant Cell Environ. 24 (2001) 253-259.
   4. Brock, T.D., Calculating solar-radiation for ecological-studies. Ecol. Model. 14, 1-19.
   5. Charles-Edwards, D.A.,Eds D Doley, GM Rimmington 'Modelling plant growth and development / by David A. Charles-Edwards, David Doley and Glynn M. Rimmington.' (Academic Press: Sydney) 1986.
   6. Collares-Pereira, M, Rabl, A., The average distribution of solar radiation-correlations between diffuse and hemispherical and between daily and hourly insolation values. Sol. Energy, 22 (1979) 155-164.
   7. de Pury, D.G, Farquhar, G.D Simple scaling of photosynthesis from leaves to canopies without the errors of big-leaf models. Plant Cell Environ. 20 (1997) 537-557.
   8. Duncan, W.G, Loomis, RS, Williams, WA, Hanau, R., A model for simulating photosynthesis in plant communities. Hilgardia, 38 (1967) 181-205.
   9. Evans, J.R., Nitrogen and photosynthesis in the flag leaf of wheat (*Triticum aestivum* L.). Plant Physiol. 72 (1983) 297-302.
   10. Farquhar, G.D, von Caemmerer, S, Berry, J.A., A biochemical model of photosynthetic
   CO_2_ assimilation in leaves of C3 species. Planta, 149 (1980) 78-90.
   11. Goudriaan, J, van Laar, H.H., 'Modelling potential crop growth processes : textbook
   with exercises.' (Kluwer Academic Publishers: Dordrecht; Boston) 1994.
3. Hammer, G.L, Wright, G.C., A theoretical-analysis of nitrogen and radiation effects on
   radiation use efficiency in peanut. Aust. J. Agri. Res. 45 (1994) 575-589.
   13. Harley, P.C, Loreto, F, Di Marco, G, Sharkey, T.D., Theoretical considerations when estimating the mesophyll conductance to CO_2_ flux by analysis of the response of photosynthesis to CO_2_. Plant Physiol. 98 (1992) 1429-1436.
   14. Leuning, R, Kelliher, FM, de Pury, D.G, Schulze, E.D., Leaf nitrogen, photosynthesis, conductance and transpiration: scaling from leaves to canopies. Plant Cell Environ. 18 (1995) 1183-1200.
   15. Monteith, J.L, Unsworth, M.H., Chapter 5 - Radiation Environment. In 'Principles of Environmental Physics (Fourth Edition).' (Eds JL Monteith, MH Unsworth.) pp. 49-79. (Academic Press: Boston) 2013.
   16. Parton, W.J, Logan, J.A., A model for diurnal variation in soil and air temperature.
   Agri. Meteorol. 23 (1981) 205-216.
   17. Wong, S.C, Cowan, I.R, Farquhar, G.D., Stomatal conductance correlates with photosynthetic capacity. Nat. 282 (1979) 424-426.

18. Yin, X., van Laar, H.H., Crop Systems Dynamics: An Ecophysiological Simulation Model for Genotype-by-Environment Interactions. Wagningen Academic Publishers, Wageningen, The Netherlands 2005.
19. Zhang, H, Nobel, P., Dependency of *C*_i_/*C*_a_ and Leaf Transpiration Efficiency on the Vapour Pressure Deficit. Funct. Plant Biol. 23 (1996) 561-568.
